# The Impact of Heterogeneity and Dark Acceptor States on FRET: Implications for Using Fluorescent Protein Donors and Acceptors

**DOI:** 10.1371/journal.pone.0049593

**Published:** 2012-11-13

**Authors:** Steven S. Vogel, Tuan A. Nguyen, B. Wieb van der Meer, Paul S. Blank

**Affiliations:** 1 Laboratory of Molecular Physiology, National Institute on Alcohol Abuse and Alcoholism, National Institutes of Health, Bethesda, Maryland, United States of America; 2 Department of Physics and Astronomy, Western Kentucky University, Bowling Green, Kentucky, United States of America; 3 Eunice Kennedy Shriver National Institute of Child Health and Human Development, National Institutes of Health, Bethesda, Maryland, United States of America; Mount Sinai School of Medicine, United States of America

## Abstract

Förster resonance energy transfer (FRET) microscopy is widely used to study protein interactions in living cells. Typically, spectral variants of the Green Fluorescent Protein (FPs) are incorporated into proteins expressed in cells, and FRET between donor and acceptor FPs is assayed. As appreciable FRET occurs only when donors and acceptors are within 10 nm of each other, the presence of FRET can be indicative of aggregation that may denote association of interacting species. By monitoring the excited-state (fluorescence) decay of the donor in the presence and absence of acceptors, dual-component decay analysis has been used to reveal the fraction of donors that are FRET positive (i.e., in aggregates)._However, control experiments using constructs containing both a donor and an acceptor FP on the same protein repeatedly indicate that a large fraction of these donors are FRET negative, thus rendering the interpretation of dual-component analysis for aggregates between separately donor-containing and acceptor-containing proteins problematic. Using Monte-Carlo simulations and analytical expressions, two possible sources for such anomalous behavior are explored: 1) conformational heterogeneity of the proteins, such that variations in the distance separating donor and acceptor FPs and/or their relative orientations persist on time-scales long in comparison with the excited-state lifetime, and 2) FP dark states.

## Introduction

Förster resonance energy transfer (FRET) is a physical phenomenon in which excited-state energy is transferred from a donor fluorophore to near-by acceptors [1]–[6]. Because the FRET transfer rate constant, *k_T_*, has an inverse sixth power dependence on the distance separating donor and acceptor fluorophores (*R_DA_*) FRET has been extensively exploited to measure separations of fluorescently-labeled sites within and between biomolecules, as well as to quantify the extent of association of interacting species [7], [8]. The recent discovery and optimization of genetically encoded fluorophores [9], [10], particularly GFP and its derivatives, has stimulated a renewed interest in FRET microscopy as a tool to measure protein-protein interactions within living cells (3, 4). In these experiments the average FRET efficiency, **〈** *E* **〉**, is measured for a population of potential donors and acceptors. While the theory of FRET is described simply for energy transfer between a single donor-acceptor pair in isolation from other donors and acceptors, analysis of FRET from a population of donors and acceptors remains problematic. The correct interpretation of FRET measurements on such populations requires a thoughtful evaluation of the physical characteristics of the fluorophores used, of the microscopic factors that influence the efficiency of energy transfer, and an appreciation of the homogeneity of these factors [2], [11].

**Figure 1:**
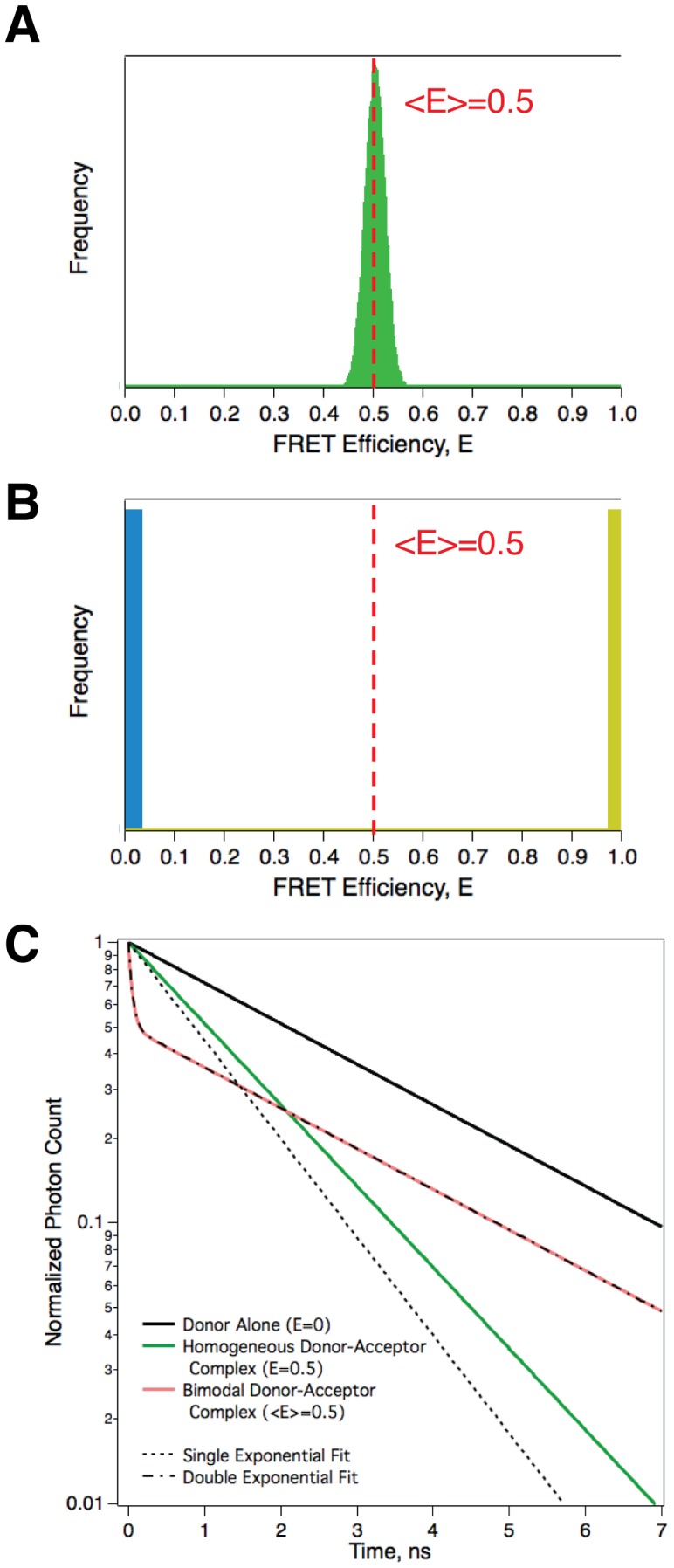
The donor fluorescence decay is an indicator of the distribution of FRET efficiency values in a population. A. Simulated distribution of FRET efficiencies for a narrow normally distributed *R_DA_* population, **〈** *E* **〉** = 0.5. **B.** Simulated distribution of FRET efficiencies for a bimodal population with E = 0 & 1. **C.** Fluorescence decays from the populations depicted in panels A and B. For comparison the decay of donors in the absence of acceptors is also plotted (Black trace). Note that the decay of the population depicted in panel B (RED trace) was poorly fit by a single exponential decay model (dotted line), but was well fit using a double exponential decay model (dashed and dots line).

In the great majority of biological FRET experiments, it is the average FRET efficiency, **〈** *E* **〉**, from a population of donors and acceptors that is measured, not individual FRET efficiencies from single donor-acceptor pairs [12]. Accordingly, if all of the individual FRET pairs in the population have rather similar values of *R_DA_*, spectral overlap integral (*J*), and dipole-dipole coupling orientation factor (*κ* ^2^), a measured **〈** *E* **〉** value might reasonably be assumed to arise from a population of similar donor-acceptor pairs with narrow, unimodally distributed *E* values. Figure 1A illustrates this condition for a unimodal population with **〈** *E* **〉** equal to 50±2%. However, if there is an appreciable degree of heterogeneity in one or more of the values of *R_DA_*, *J*, and *κ* ^2^ then the population of donor-acceptor pairs may have the same **〈** *E* **〉** but arising from a more complicated distribution. In figure 1B, an example of a bimodal distribution of *E* values is depicted. It is a distribution which would apply, for example, to a heterogeneous binding system in which half of a donor-containing population is bound to an acceptor-containing partner such that the acceptor is far away from the donor, and/or their mutual orientations are unfavorable to FRET (individual donor-acceptor pairs have *E* values at or near 0; Blue), the other half bound to an acceptor-containing partner with favorable FRET conditions, small donor-acceptor separations with favorable mutual orientations (Yellow). Less extreme cases where the donor-acceptor pairs equally form two different types of complexes, **〈** *E*_1_ **〉** = 0.3 and **〈** *E*_2_ **〉** = 0.7, for example, would also produce the same **〈** *E* **〉** (not depicted). Obviously, an **〈** *E* **〉** measurement alone cannot differentiate between these distributions. In contrast, the fluorescence decay of the donor emission from the population depicted in panel A will exhibit very close to mono-exponential decay (Fig. 1C, GREEN trace), whereas that of the donors in the population depicted in panel B will closely approximate a double exponential (RED trace). Even though the underlying distribution of microscopic FRET efficiencies is not known from a population FRET efficiency measurement, FRET measurements based on donor fluorescence lifetime determinations, either in solution samples or by fluorescence lifetime imaging of cells under the microscope (FLIM), can be used to detect evidence for heterogeneity [13].

Excited-state decay studies of complexes composed of a single donor-acceptor FRET pair of (different) donor and acceptor fluorescent proteins (FPs) tethered to each other by an amino-acid linker and expressed within cells have recently been characterized in our laboratory [14] and elsewhere [13]. Since each of these constructs comprises one donor and one acceptor separated by a short amino-acid linker, the donor emission is expected to decay as a single exponential, but this is not observed [13]. For example, C5V, is a FRET standard developed in our laboratory, composed of a blue FP donor (Cerulean) covalently linked to a yellow FP acceptor (Venus) via a 5 amino-acid linker [14], [15]. When expressed in cells, this construct has a FRET efficiency of 43±2% as measured by 3 different techniques [14], [15]. A single exponential model did not describe adequately the C5V decay, but the data statistically was fit well using a double exponential model. Frequency-domain FLIM measurements have also confirmed that C5V decays with multiple components [16]. One explanation for this complex decay behavior is that many, if not all, FP donors, even in the absence of acceptors, fail to decay as a single exponential [17]–[19]. Nonetheless, even when GFP derivatives engineered to decay as a single exponential in the absence of acceptors were used to build tandem FP FRET pairs, their decay in the presence of an acceptor still had multiple components [13], [14].

Heterogeneity in any or all of the values of *R_DA_*, *J*, and *κ* ^2^ in a FRET system may result in non-trivial donor decay complexity whose decay components are not easily assigned. Complexity in donor decay in the absence of acceptor (which would itself give rise to complexity in the presence of acceptor even in the absence of heterogeneity in the FRET parameters) will add a further layer of uncertainty to the assignment of decay components. Thus, before dual-component decay analysis can be used to reveal the fraction of donors that complex with acceptors in FRET experiments, the anomalous decay behaviors of donors alone and in tandem FRET pairs must first be understood. In the following, Monte Carlo simulations and analytical models are used to explore how the observed complex decay behavior of FP FRET pairs might arise, specifically in the idealized case that the same FRET donor in the absence of acceptor decays as a single exponential.

### FRET Theory

FRET is a non-radiative process mediated by dipole-dipole interactions. For appreciable levels of FRET to occur, three conditions must be met. First, the distance between the donor and the acceptor (*R_DA_*) must be short, typically in the range of 1 to 10 nm [7]. For a single donor-acceptor pair in a given configuration, the transfer rate constant, *k_T_*, depending, as shown both theoretically [6] and experimentally [7], on the inverse sixth-power of the separation, is given by:
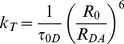
(1)where 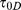 is the excited-state (fluorescence) lifetime of the donor molecule defining its mono-exponential decay in the absence of acceptors, and *R*_0_ is the Förster distance, the separation, specific for a particular donor-acceptor pair, at which 50% of the donor excitation events result in energy transfer to the acceptor. The value of *R*_0_ is given by:
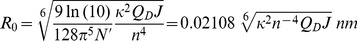
(2)where N’ is Avogadro’s number per mmole, 6.022×1020, n the refractive index of the nearby medium [20], 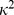 the orientation factor for dipole-dipole coupling, which in R0 calculations is formally taken to have a value of 2/3 corresponding to its dynamic random average value, 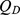 the fluorescence quantum yield of the donor in absence of the acceptor, and the numerical factor 0.02108 applying when the overlap integral J is defined in units of nm4cm2/mmole.

The efficiency of energy transfer, *E*, is then defined by the ratio of the transfer rate constant to the total rate of deactivation of the fluorophore:
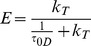
(3)


Substitution from Eq 1 for 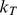
reveals that *E* does not depend *per se* on the donor lifetime, only on its ratio to the excited-state lifetime:
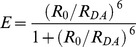
(4)When *R_DA_* is equal to *R*_0_, *k_T_* = 1*/τ*_0*D*_ and *E* = 0.5. Note that, due to the sixth-power dependence of *k_T_*, variation in the separation *R_DA_* can dramatically alter the FRET efficiency. For example, when *R_DA_* is twice the *R*_0_ value *E* will be less than 1.6% and, similarly, when *R_DA_* is half the *R*_0_ value *E* will be greater than 98.4%.

A second requirement for FRET to occur is that the loss of energy from the donor as it transitions from an excited-state electronic energy sub-level to a ground state sub-level must be equivalent to the energy absorbed by an acceptor when it, in parallel, transitions from a ground-state to an excited-state sub-level. Compliance with this requirement for any particular pair of donors and acceptors is confirmed by the overlap of donor emission and acceptor absorption spectra and is quantitatively determined using the spectral overlap integral *J* (eq 2), defined as the product of these two overlapping spectra weighted by the fourth power of the wavelength and normalized to the abundance of all excited states of the donor [6]:
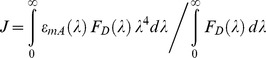
(5)where 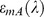 is the molar extinction coefficient of the acceptor and 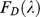 the donor fluorescence intensity per unit wavelength interval, at wavelength 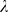. The overlap integral influences *k_T_* and *E* by virtue of its effect on the value of *R*_0_ (Eq 2). The greater the overlap of these two spectra, and the longer the wavelengths involved, the greater is the probability that FRET will occur. Thus, if there is no spectral overlap at all, *J* = 0 so that *R*_0_ = 0 (Eq 2), *k_T_* = 0 (Eq 1), and *E* = 0 (Eqs 3 and 4).

From Equation 2 it is clear that other factors can potentially influence the value of *R*_0_ and thus affect the FRET efficiency. The quantum yield of the donor and refractive index of the adjacent medium [20] are typically assumed to be homogeneous and constant. For example, because their fluorescent moieties are shielded from most environmental influences within the surrounding β-barrel structure, the quantum yields of FPs – and *pari passu*, their excited-state decay behavior – are unlikely to exhibit any significant variation from molecule to molecule in a given construct [21]. However, these fluorophores are known to ‘blink’ between normal and dark states [22]–[24], which due to partial population losses of either or both transfer partners, will affect the measurement of E values obtained to an extent dependent on the method of measurement.

Finally, a third requirement for FRET is that the orientation of the transition dipoles of the donor and acceptor not be perpendicular to one another, but more generally that the absorption dipole of the acceptor not be perpendicular to the electric field of the excited donor [25]–[27] since, in such configurations, the absorption dipole of the acceptor cannot couple to the oscillating electric field produced by the excited donor. The dipole-dipole coupling orientation factor (*κ* ^2^) can be expressed in terms of this field coupling by [25], [26], [28]:
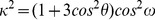
(6)where 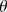 is the angle between the donor emission dipole orientation and the donor-acceptor` separation vector, and ω is the angle between the donor electric field vector at the acceptor location and the acceptor absorption dipole orientation. Since 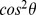 and 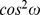 can only have values ranging from 0 to 1, *κ* ^2^ is limited to values between 0 and 4. No donor emission dipole orientation can, of and by itself, reduce the FRET efficiency to zero, which requires that the acceptor absorption dipole orientation is perpendicular to the donor electric field vector, *ω* =  *π*/2, 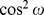 = 0, *κ* ^2^ = 0, *R*_0_ = 0, *k_T_* = 0 and *E* = 0. Again, should this orientation be close to perpendicular, and for a random distribution, the probability of this is very high relative to that for orientations far from perpendicular, then the FRET efficiency may be negligible even for separations well below the *R*_0_ value. Because the relevant dipole orientations are rarely known, either an appropriate *κ* ^2^ value is assumed, or some limits set upon its possible values [25], [26], [29], [30], though such information may sometimes be available from crystallographic (or possibly nuclear magnetic resonance) structures and/or through the application of molecular dynamics calculations, either *ab initio* or, possibly, in conjunction with NMR structures, or, as has been advocated by Truong and coworkers [31], [32] and recently applied in a study of IgE conformational change [33], in conjunction with crystallographic information.

## Results

### Separation Heterogeneity can Produce Bimodal FRET Efficiency Distributions

**Figure 2:**
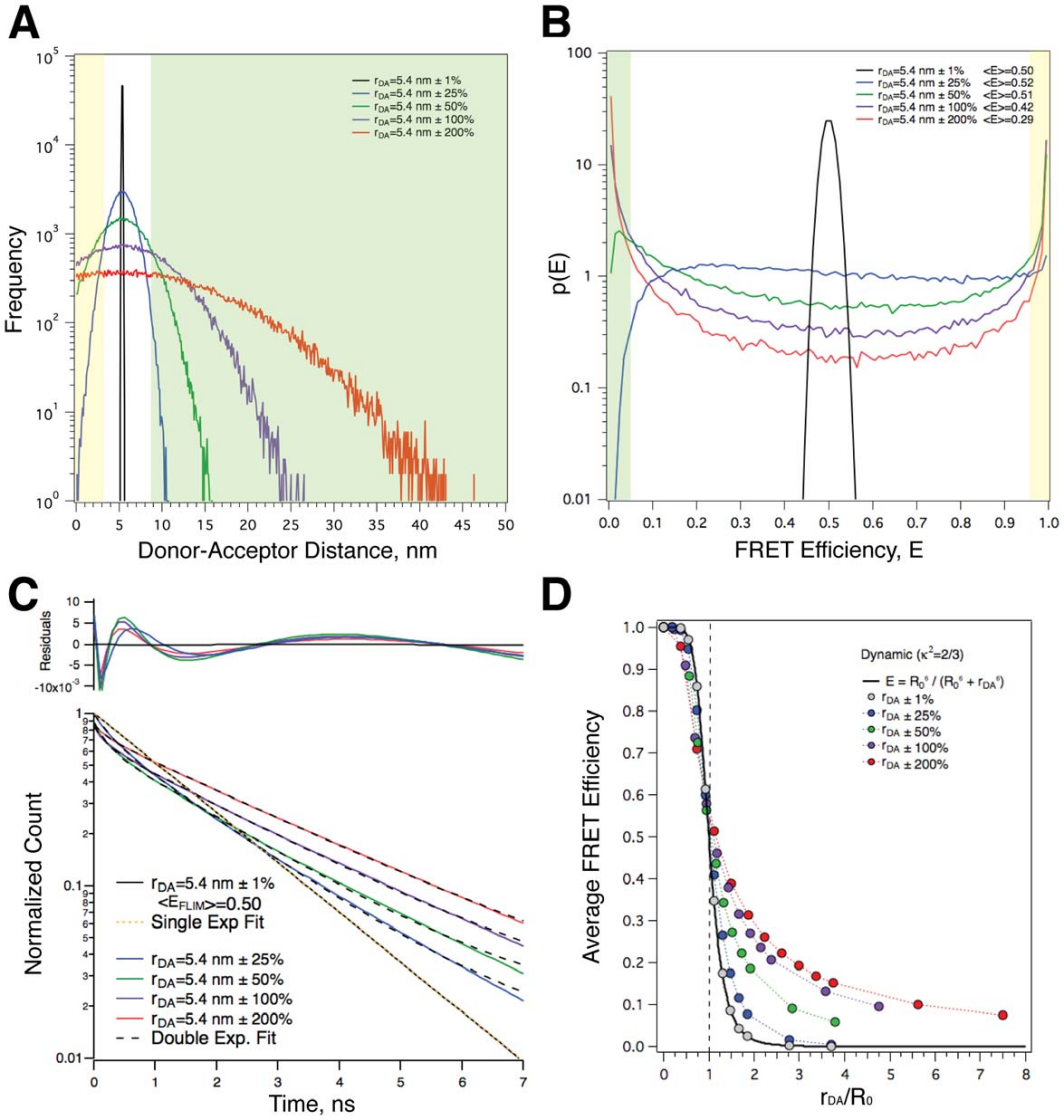
Large variance in separation (*R_DA_*) produces bimodal FRET efficiency probability density histograms, multi-exponential decays, and alters the FRET efficiency from that pertaining to the mean separation. **A.** A histogram of *R_DA_* values from 5 Monte-Carlo simulations, each having a Gaussian distribution with its mode at 5.4 nm, but with standard deviations ranging from 1 to 200% of the mode. Note that points with negative separation were dropped from the distribution in the simulation. **B.** The distribution of FRET efficiency probabilities from the populations depicted in panel A. The *R*_0_ value was set to 5.4 nm, the lifetime of the donor in the absence of acceptors was set to 3 ns, and *κ* ^2^ was set to 2/3 to simulate the dynamic random isotropic reorientational regime. green tinted area represents *E*<0.05, and the yellow tinted area represents *E*>0.95. These points corresponded to the *R_DA_* values in panel A with similar tints. **C.** Fluorescence decays from the populations depicted in panel A. D. The *R*_0_ normalized dependence of 〈*E*〉 on both *R_DA_* and its variance.

Monte-Carlo simulations were used to investigate the impact of separation distance heterogeneity on FRET efficiency and lifetime measurements (Fig. 2). For each simulation, 100,000 *R_DA_* values were randomly generated from Gaussian distributions centered at 1 to 20 nm. To simulate *R_DA_* heterogeneity the standard deviation of each Gaussian was increased from 1 to 200% of the center value. Negative *R_DA_* values were dropped. A histogram showing a subset of our simulations with *R_DA_* values set to a 5.4 nm center value and increasing *R_DA_* heterogeneity is plotted in figure 2A.

Individual *R_DA_* values were transformed into *k_T_* values using Eq 1 with a *τ_0D_* value of 3 ns (the Cerulean lifetime) and an *R*_0_ of 5.4 nm (containing the dynamic random average value of 2/3 for *κ* ^2^) to model Cerulean to Venus energy transfer [34] and the *k_T_* values transformed into *E* values using Eq 3. Histograms approximating the probability density *p*(E) of FRET efficiencies for the populations depicted in panel A, which represent their distribution arising from the distribution of separations, are shown in figure 2B. As expected, with *R_DA_* values near the *R_0_* value, a distribution with small variance (1%, BLACK trace) was centered around a FRET efficiency of 0.5, with an average FRET efficiency, **〈** *E* **〉**, very close to 0.5. As the standard deviation of the Gaussian *R_DA_* distributions increased, the distribution of FRET efficiencies broadened, and is transformed from a distribution with a single peak into a bimodal distribution where the most abundant *E* values occur closer and closer to 0 (no FRET) or 1 (100% FRET) with higher and higher probability. The green and yellow tinted regions of panel B represent the fraction of the population that has *E* values under 0.05 or over 0.95 respectively. The sub-set of *R_DA_* values that generated these extreme *E* values are shown in the corresponding green and yellow tinted regions depicted in panel A. Note that **〈** *E* **〉** for the 25% standard deviation *R_DA_* population is also near 0.5 despite the dramatic differences in the microscopic FRET efficiency distribution. In contrast, **〈** *E* **〉** was significantly lower than 0.5 for the 50%, 100% and 200% standard deviation populations.

### Separation Heterogeneity can Produce Multi-exponential Decays

Ensemble fluorescence decay curves were calculated for the populations depicted in panel A using Equation 7:
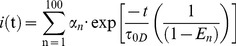
(7)where 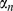 are precisely the frequency values for the individual FRET efficiency bins displayed in figure 2B, and *E_n_* are the FRET efficiency values centered on each of the 100 bins, while *τ*_0*D*_ is the fluorescence lifetime of the donor in the absence of acceptors (corresponding to the excitation-weighted average of 3 ns for Cerulean). Ensemble decay curves are shown in figure 2C. Decay curves were fit to either a single or double exponential decay model. The ensemble decay curve for an *R_DA_* = 5.4 nm ±1% Gaussian population was well described using a single exponential decay model with a decay time of 1.5 ns corresponding, as expected, with the quantum yield of 0.50 and a lifetime in absence of acceptor of 3.0 ns. All of the other *R_DA_* = 5.4 nm populations with standard deviations ranging from 25–200% were poorly fit by a single exponential decay model. While these distributions were better fit using a double exponential decay model, these too were inadequate, as can be seen both directly in the decay plots and more definitively in the weighted residuals displayed in figure 2C.

### Increasing Separation Heterogeneity around a Mean Separation Alters 〈*E*〉

Monte-Carlo simulations as depicted in figures 2A and 2B were also performed for *R_DA_* values ranging from 1 to 20 nm. In figure 2D, the **〈** *E* **〉** from these simulated populations are plotted as a function of the average separation of the population for 1, 25, 50, 100 and 200% standard deviation data sets. For comparison the dependence of **〈** *E* **〉** on *R_DA_* for FRET pairs with a fixed separation distance based on Förster theory (again assuming a *κ* ^2^ value of 2/3) is also plotted. While the average FRET efficiencies for the *R_DA_* ±1% populations were indistinguishable from those for a single fixed pair, there were significant deviations from this for the 25, 50, 100, and 200% standard deviation populations. In general, for these conditions, lower than expected **〈** *E* **〉** values were observed for *R_DA_* values below *R_0_* (vertical dashed line) and larger than expected **〈** *E* **〉** values were observed for *R_DA_* values above *R_0_*.

### Long-lived Acceptor Dark States can also Produce Bimodal FRET Efficiency Distributions

**Figure 3:**
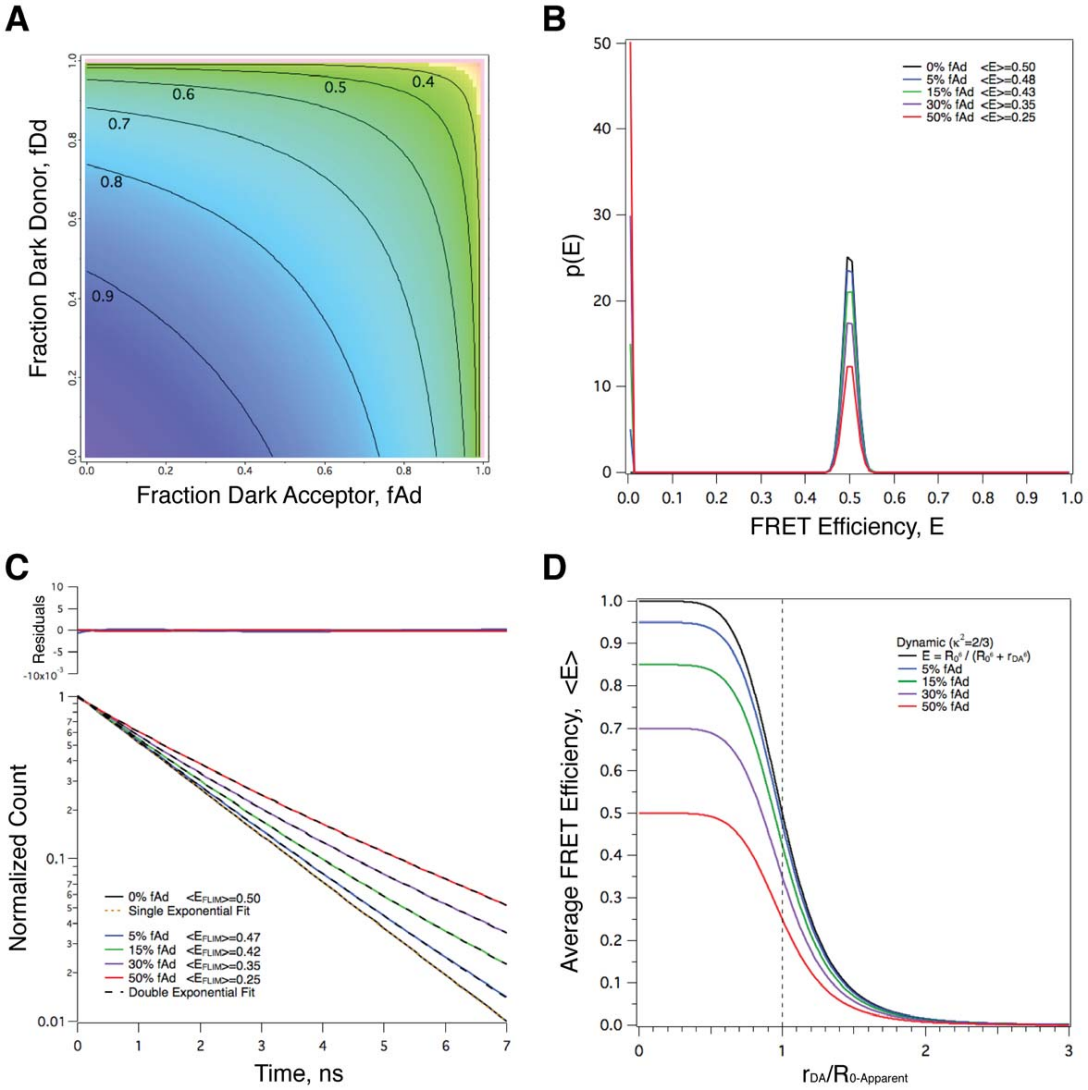
Long-lived acceptor dark states can produce bimodal FRET efficiency distributions, multi-exponential donor excited-state decays, and a decrease in measured average FRET efficiency compared with that for the mean separation. **A.** Long-lived dark states (blinking and flickering) in donor and/or acceptor fluorophore populations may attenuate the apparent *R*_0_ value for FRET, in the former by reducing the measured quantum yield, in the latter by reducing the measured extinction coefficient and thereby the spectral overlap integral. The attenuation factor (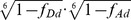) is plotted as a function of dark donor fraction (*f_Dd_*) and dark acceptor fraction (*f_Ad_*). **B.** Monte-Carlo simulations were used to model the distribution of FRET efficiencies from populations with 0 to 50% acceptor dark states. Separations were modeled using a Gaussian with a standard deviation equal to 1% of the mode. The true *R*_0_ was fixed for all samples at a value of 5.4 nm, the lifetime of the donor in the absence of acceptors set to 3 ns, *κ^2^* set to 2/3, and it was assumed that dark states do not absorb in the region of donor emission. **C.** Fluorescence decays for the populations depicted in panel B. **D.** Dependence of measured 〈*E*〉 on the ratio of fixed (i.e., no distribution width) *R_DA_* to true *R*_0_ and the fraction *f_Ad_* of acceptors in the dark state.

A photo-physical behavior called ‘flicker’ or ‘blinking’ has been observed in several fluorescent proteins and in some organic dyes [23], [24], [35]. These fluorophores exhibit reversible transitions between a ‘bright’ fluorescent state and a non-fluorescent ‘dark’ state. The rate of transition between these states for FPs occurs on a time scale of microseconds to seconds, and the fraction of molecules in the dark state can be as high as 30–60% [23], [24], [35]. As the blinking rate of fluorescent proteins is typically orders of magnitude slower than their fluorescence decay rates, FP donors and acceptors can be thought of as being in *either* their bright *or* their dark state, with negligible probability of transition from one to the other while the “bright” donor is in its excited state. In a FRET experiment, both donor and acceptor fluorophores will be influenced by blinking behavior. Vis-à-vis FRET donors, blinking behavior may alter the quantum yield of the donor (*Q_D_*) and possibly also the extinction coefficient of the acceptor, therefore artifactually changing the value of *R_0_* (Eq 2; see Fig. 3A). Nonetheless, blinking behavior for a FRET donor should have no significant impact on FRET measurements because *R_0_* values used in FRET calculation most likely have already incorporated the impact of blinking when they were determined. In contrast, blinking behavior for FRET acceptors potentially can have a dramatic affect on FRET efficiencies. If dark-state occupancy prevents fluorophores from acting as FRET acceptors, donors in close proximity to dark-state acceptors will not be able to transfer energy by FRET; this will result in a reduced **〈** *E* **〉**. To investigate the potential impact of blinking behavior on **〈** *E* **〉** measurements, as well as on the lifetime, Monte-Carlo simulations were performed as described for figure 2, using donor-acceptor pair populations with *R_DA_* values distributed with ±1% standard deviation, and with acceptor blinking behavior where either 0, 5, 15, 30 or 50% of the acceptors are in a dark state preventing them from acting as FRET acceptors (Fig. 3). In the absence of blinking the FRET efficiency distribution (i.e., *p*(E), the probability density) for a population with *R_DA_* values of 5.4±1% was, within the noise level of the Monte-Carlo simulation, symmetrical about a value of 0.5, which is also the mean over the distribution (Fig. 3B, BLACK trace). As the fraction of FRET pairs with dark-state acceptors (*F_D_*) increased from 0 to 50% (BLUE, GREEN, PURPLE and RED traces) the amplitude of the peak at *E* = 0.5 decreased proportionally to the bright-state fraction (1−*F_D_*). Concomitant with this decrease, the frequency of FRET pairs with *E* = 0 increased, and this increase was also directly proportional to *F_D_*. Thus, the FRET efficiency distributions in the presence of blinking were bimodal with peaks at *E* = 0 and 0.5, and the relative numbers of molecules in these two states varied reciprocally as a function of *F_D_*. The average FRET efficiencies for populations with acceptor blinking, **〈** *E*_B_ **〉**, decreased proportionally with the fraction of acceptors in the bright state:
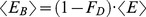
(8)where **〈** *E* **〉** is the average FRET efficiency in the absence of blinking.

### Acceptor Dark States can also Produce Multi-exponential Donor Excited-state Decays

The ensemble fluorescence decays for the populations depicted in figure 3B were calculated as described for figure 2C, and are plotted in figure 3C. In the absence of blinking the lifetime was described well with a single exponential decay model, while in the presence of blinking the lifetimes were described well with a double exponential decay model approximating a fraction *F_D_* of the lifetime in absence of acceptor together with a fraction (1−*F_D_*) of the lifetime obtained in the absence of blinking. Apparent average FRET efficiencies were calculated for these distributions based on their excitation-weighted average decay times, and agreed well with the average FRET efficiencies calculated from the microscopic FRET efficiencies depicted in panel B.

### The Existence of Acceptor Dark States Alters the Dependence of Measured Efficiency 〈E_B_〉 on Separation

Equation 8 can be used to calculate the impact of acceptor blinking on the relationship between separation and measured efficiency **〈**E_B_ **〉** for any value of *F_D_*. This is depicted in figure 3D for FRET pairs with a fixed separation. Figure 3D summarizes the impact of acceptor dark states on average FRET efficiency as a function of separation normalized to the characteristic Förster separation *R*_0_.

### Monte Carlo Simulation of the *κ* ^2^ Distribution in an Isotropic Population

**Figure 4:**
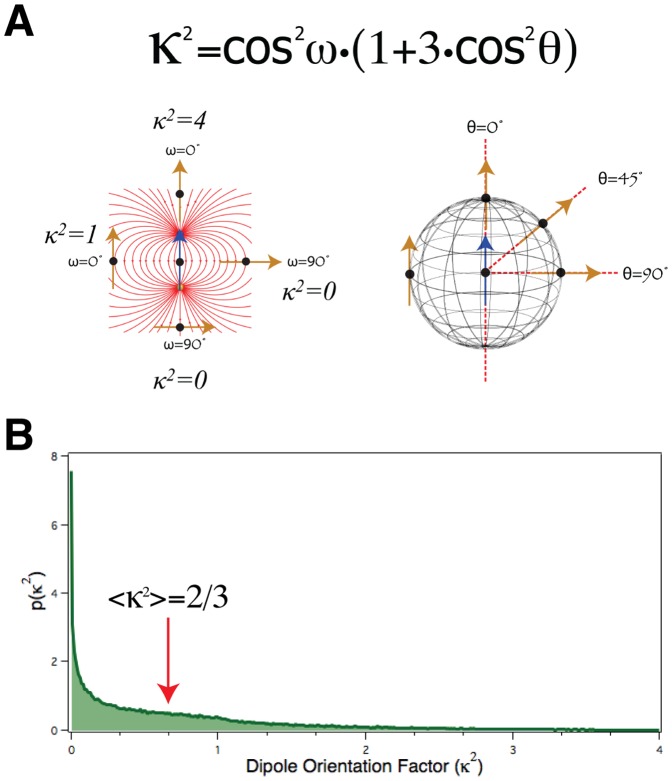
Monte Carlo simulations of isotropic *κ* ^2^ **distribution. A.** Cartoon illustrating how the dipole orientation factor *κ* ^2^ is based on 2 angles, *θ* and *ω* (see Eq 6). **B.** Monte Carlo simulation of the *κ* ^2^ probability distribution assuming that *θ* and *ω* are randomly distributed (isotropic). Note that the mode of this distribution is 0 and the average is 2/3.

The dipole orientation factor, *κ* ^2^, is usually assumed to have a value of 2/3 in FRET experiments, and this value is almost always assumed when *R*_0_ values for specific donor-acceptor pairs are calculated. Because *κ* ^2^ may have any value ranging from 0 to 4, FRET transfer rate constants calculated using equation 1 may in reality have any value ranging from 0 to 6 times the calculated value based on the *κ* ^2^ = 2/3 assumption. To perform Monte-Carlo simulations to evaluate the impact of this assumption on the distribution of FRET efficiencies in populations, it is necessary first to understand the rational for using *κ* ^2^ = 2/3. The dipole orientation factor *κ* ^2^ is most compactly expressed as a function of 2 angles, *θ* and *ω* (equation 6). The position and orientation of the emission dipole of a donor fluorophore can be envisioned as a blue arrow, and the position and orientation of the absorption dipole of an acceptor fluorophore as a yellow arrow (Fig. 4A). The red lines connecting the two ends of the blue arrow on the left side of panel A depict the orientation in space of the electric field lines created when the donor is in the excited state. The angle formed between the yellow arrow and the field line at its location is the angle *ω*. Two examples each for when *ω* is equal to 0° (parallel to the electric field) or 90° (perpendicular) are shown on the left side of panel A. If the acceptor is randomly oriented in space then *ω* can have any value from 0° to 90° and therefore cos^2^ *ω* can have any corresponding value from 1 to 0. The angle formed between the orientation of the blue arrow and the line connecting the donor to the acceptor (dashed red lines) defines the angle *θ* (Fig. 4A left). Note that the orientation of the acceptor absorption dipole does not influence the value of *θ*. For different locations of the acceptor around the donor in three-dimensional space, *θ*, like *ω*, can have any value from 0°–90° and therefore cos^2^ *θ* can have any corresponding value from 1–0. It is also noted that, even if it is assumed that the orientation of the acceptor is randomly distributed in three dimensions it is much more likely that *ω* and *θ* will have values closer to 90° than to 0°. This is an unsettling aspect of isotropic (random three-dimensional) angular distributions.

Monte-Carlo simulations were used to model the distribution of *κ* ^2^ values from a population of donor-acceptor pairs in which the orientations of donor and acceptor are independently isotropically distributed. First, a random number generator was used to produce a set of random angles from isotropic distributions. Next, random pairings of these values were used to generate *ω* and *θ* values. These were then used to calculate a population of *κ* ^2^ values using Eq 6. A histogram of this population of *κ* ^2^ values was generated, and normalized to the total number of values calculated (i.e., the integral of the histogram was set equal to unity) to produce a plot approximating the probability density for the orientation factor *p*(*κ* ^2^) for an isotropic population as shown in figure 4B. This probability distribution generated by Monte-Carlo simulation was indistinguishable from similar plots generated by closed-form calculations [26], [27], [36]. Notice that the most probable value for *κ* ^2^ is 0 and the least probable is 4. Also notice that the average value of *κ* ^2^ from this distribution is 2/3 (RED arrow). The use of the average value of 2/3 for *κ* ^2^ in FRET calculations corresponds physically to the tacit assumption that the relative orientations of the donor and acceptor dipoles are either fixed in such a configuration as to generate this value, or is the dynamic random average corresponding physically to independent reorientation, as a result of rotational diffusion (and/or to transition moment degeneracy) of donor and acceptor dipoles within the donor-acceptor framework (i.e., wholesale rotation of the frame itself has no effect on the orientation factor) over random isotropic (or pseudo-isotropic, i.e., hemispherical) distributions, which is rapid with respect to the rate of energy transfer: the *dynamic random isotropic averaging regime*. As most organic dyes have rotational correlation times in solution on the ps time scale, while the same dyes have fluorescence lifetimes on the ns time scale, the use of an average value of 2/3 for *κ* ^2^ is justified and can be used if rotational motion allows all orientations to be sampled randomly in a time shorter than the inverse transfer rate. The situation for FRET with donor and acceptor FPs is quite different. As a result of their high molecular weight, free FPs rotate more slowly in low-viscosity aqueous solutions, having rotational correlation times in the range of 15–20 ns [35], [37]–[41] whereas their fluorescence lifetimes are typically much shorter, in the range of 2–4 ns. When FPs are engineered into other proteins, they may exhibit segmental flexibility with even slower rotation, depending on the size of the flexible polypeptide segment within which they are contained and the viscosity conditions in their local environment, but it will certainly not be faster. Thus, during the donor excited state lifetime, the relative orientations of FP absorption and emission dipoles will essentially not change. Should the rotation be isotropic (or pseudo-isotropic), the system is in the *static random isotropic averaging regime*, and in this situation the use of a *κ* ^2^ value of 2/3 is completely unjustified [25], [26], [33], as explored in detail below.

### For a Population with the Same Distribution of Separations, Dynamic and Static Random Isotropic Regimes have Different FRET Efficiency Distributions and Averages

**Figure 5:**
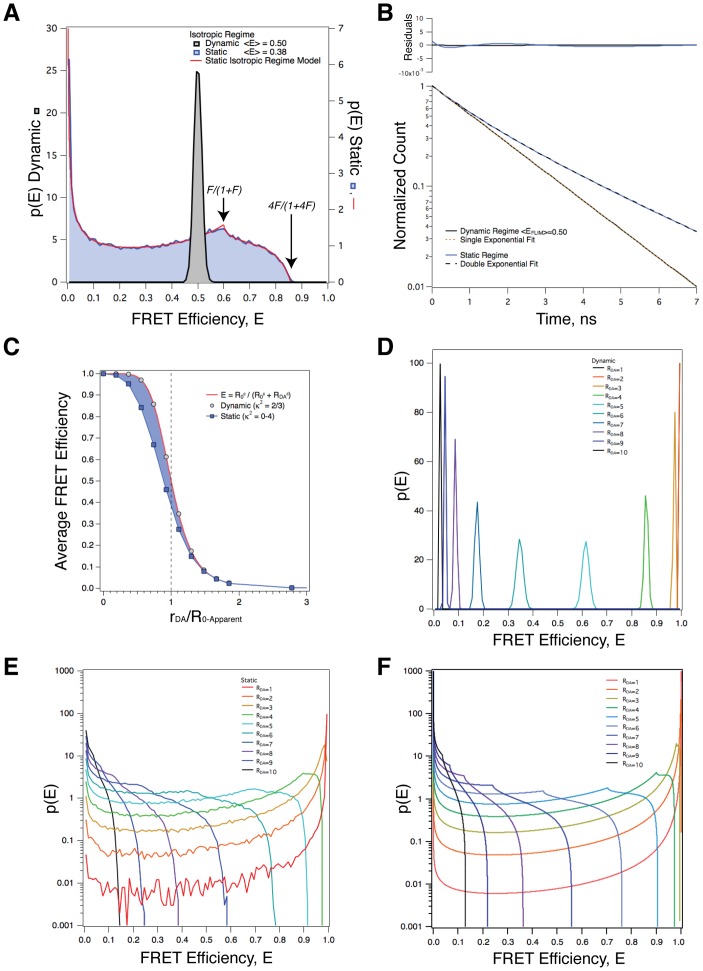
For the same separation distance, dynamic and static isotropic regimes have different FRET efficiency distributions and decays. **A.** The FRET efficiency distributions from Gaussian populations with a mean *R_DA_* value of 5.4 nm±1% in either the dynamic random isotropic reorientational regime (*κ* ^2^ = 2/3, GRAY peak) or the static random isotropic orientational regime (BLUE bimodal distribution). **B.** Fluorescence decays for the populations depicted in panel A. **C.** The dependence of 〈*E*〉 on *R_DA_* in these dynamic (GRAY open circles) and static (BLUE squares) regimes. The blue area between these curves depicts the region between the dynamic and static regimes into which the FRET efficiencies for samples that have rotational correlation times similar to the inverse of the energy transfer rates will fall. **D** and **E.** FRET efficiency distributions (probability densities, *p(E)*) used to generate the dynamic (D) or static (E) average FRET efficiency curves displayed in panel C. Note that in the static random isotropic regime, samples tend to have FRET efficiencies either near 0% or centered at *F*/(1+*F*). **F.** FRET efficiency distributions, *p(E)*, for fixed separation ranging from 1 to 10 nm and a Förster separation of 5.4 nm (*F*-values ranging from 3.7×10^4^ to 3.7×10^−2^) calculated analytically.

To compare the expected distribution of FRET efficiencies for a population of FRET pairs exhibiting random isotropic distributions of donor and acceptor dipoles in the dynamic and static reorientational regimes, Monte-Carlo simulations were used to generate a population of *κ^2^* values as described above, as well as a normally distributed population of separations (*R_DA_*) with a central value of 5.4 nm and 1% *σ_R_*, as previously described. For the dynamic regime, an average transfer rate constant **〈** *k_T_* **〉** is defined by:
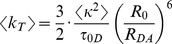
(9)where **〈** *κ^2^* **〉** has its dynamic random isotropic average value of 2/3, and which applies to each individual *R_DA_* in the distribution. This calculation is equivalent to that which would be obtained using, as in all of the previous simulations, equation 1 to transform *R_DA_* values into **〈** *k_T_* **〉** values. Equation 9 implicitly contains the average of the orientation factor for this regime within *R*_0_ as **〈** *κ^2^* **〉** = 2/3. For the static regime simulation, the separations and values from the distribution of orientation factors were randomly associated and used to generate a population of FRET transfer rate constants from:
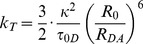
(10)where the factor of 3/2 in this equation removes the influence of the 2/3 κ2 value embedded in R0, and the influence of the static κ2 regime is now represented by the inclusion of a specific κ2 value randomly selected from a population similar to the one that generated the κ2 probability density histogram depicted in figure 4B. Next, the two transfer rate constant populations corresponding to these static and dynamic averaging regimes were transformed into populations of FRET efficiencies using equation 3. The distribution of FRET efficiencies for the dynamic and static isotropic populations, using a bin resolution of 0.01, is plotted in figure 5A. While the dynamic population is narrowly and symmetrically distributed around a peak FRET efficiency value of 0.5, the static distribution is broad and bimodal with a sharply reached maximum within the efficiency binned between E = 0 and E = 0.01, and a smaller peak at E = ∼0.6. The average efficiency 〈E〉 for the dynamic regime population, whose distribution is actually slightly asymmetric, is 0.50 with a standard deviation of 0.01, while the static isotropic regime population is characterized by a very broad asymmetric distribution with 〈E〉 = 0.38 and a very large standard deviation of 0.25 reflecting that. This distribution was similar to probability density distributions used to analyze expected inter-dye distances for polyprolines in glycerol [42] based on calculations presented by Dale and colleagues [25].

### An Analytical Solution for the Distribution of FRET Efficiency Values for a Population of FRET Pairs in the Static Isotropic Regime

An analytical solution for the probability density *p(E)* of FRET efficiencies in the static isotropic regime was derived (see Methods) and has 3 phases, the first of which, describing the probability of having a FRET efficiency, E, falling between zero and *F*/(1+*F)*, is defined by Eq 11:
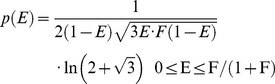
(11)where *F*, named in honor of Theodor Förster, is defined as:



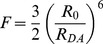
(12)Note that for this first phase the most probable FRET efficiency is zero. The second phase, describes the probability of having FRET efficiencies between *F*/(1+*F*) and 4*F*/(1+4*F*) and is described by Eq 13:
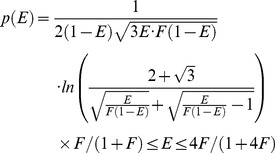
(13)


Note that this second phase has a peak at *F*/(1+*F*) and decreases to its lowest probability (zero) at 4*F*/(1+4*F*). The third phase describes the probability of having a FRET efficiency greater than 4*F*/(1+4*F*): *p*(*E*) = 0 in the third phase. Equations 11 and 13 were used to calculate the FRET efficiency probability density for a static isotropic population whose *R_DA_* value is equal to *R*_0_. This function is plotted in figure 5A (Smooth RED line). When *R_DA_ = R*_0_, *F* = 1.5, *F*/(1+*F*) = 0.6, and 4*F*/(1+4*F*) = 0.86.

### Different Donor Fluorescence Decays are Expected for Populations of FRET Pairs with Random Isotropic Orientational Distributions when they are in the Dynamic or Static Isotropic Regimes

The ensemble fluorescence decays for the populations described in figure 5A were calculated as described for those displayed in figure 2, and are plotted in figure 5B. In the dynamic reorientational regime the lifetime fitted well to a single exponential decay model, while in the static regime the lifetime required a double exponential decay model. Apparent average FRET efficiencies 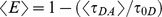 were calculated for these distributions based on their average decay time
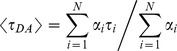
where 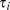 are the lifetimes obtained from the energy transfer efficiency probability density histograms depicted in figure 5A and given by



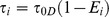
in which 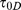 is taken as 3 ns, and the 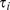 correspond with the 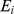 values at the center of the bins (of width 0.01, corresponding to lifetime bin widths of 0.03), while 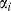 are the corresponding binned efficiency probability densities. These average FRET efficiencies, 0.5 and 0.37, were similar to those of 0.5 and 0.38, respectively, calculated from the microscopic FRET efficiencies depicted in figure 5A.

### The Dependence of 〈*E*〉 on Separation is Different for Dynamic and Static Random Isotropic Orientational Regimes

Monte-Carlo simulations as depicted in figure 5A were also performed for *R_DA_* values ranging from 1 to 20 nm. In figure 5C, **〈** *E* **〉** from these simulated populations are plotted as a function of average separation of the population for the dynamic (open circles) and static (dark blue squares) regime data sets. For comparison the dependence of FRET efficiency on separation for FRET pairs with a fixed separation is also plotted (red solid line). While **〈** *E* **〉** for the *R_DA_*±1% dynamic isotropic regime populations were indistinguishable from that for the dynamic regime, fixed pair, there were significant differences from this for the static regime populations. In general, lower than expected **〈** *E* **〉** values were observed. These differences (for a FRET pair with a *R*_0_ value of 5.4 nm) were most profound in the *R_DA_* range of 1.2 to 7 nm, i.e. between about 0.22 and 1.30 on the abscissa scale of figure 5C. The area depicted in blue between the dynamic and static curves represents the **〈** *E* **〉** values expected for a population in which the rotational correlation times of the donor and/or acceptor are commensurate with the excited-state lifetime of the donor in the presence of acceptor.

### Monte Carlo Simulations and an Analytical Solution for the Probability Density of FRET Efficiency in the Static Isotropic Regime Produce Closely Similar Results

FRET efficiency probability density histograms generated by Monte-Carlo simulations, used to calculate the ensemble average FRET efficiencies plotted in Figure 5C, are shown for dynamic (Fig. 5D) and static (Fig. 5E) isotropic reorientational regimes for separations ranging from 1 to 10 nm. As expected, **〈**E**〉** values for the dynamic regime plotted in Figure 5C (open circles) matched the peak microscopic FRET efficiencies observed in Figure 5D. In contrast, for the static regime, microscopic FRET efficiencies all exhibited irregular distributions with a peak in the interval closest to zero FRET efficiency, even when the separations were much smaller than the Förster distance, *R_0_*. These distributions had either an inflection point or a secondary peak at intervals within which the efficiency had a value corresponding to *F*/(1+*F*). FRET efficiency probability densities calculated using equations 10 and 12, to model samples with the same *R_0_* (5.4 nm) and fixed separations ranging from 1–10 nm, are plotted in figure 5F. These distributions exhibit closely similar contours to those generated by our Monte-Carlo simulations (Fig. 5E) but they are sharper (note the very definite inflection points or peaks occurring at *F*/(1+*F*) for the probability density) and differ substantially near FRET efficiencies of 0 and 1 due to the binning of the simulation results in intervals of *E* not small enough to approximate the densities due to their rapid changes in those regions.

## Discussion

The accurate interpretation of ensemble FRET measurements requires an understanding of the underlying distribution of microscopic FRET efficiency values within the population. In most FRET experiments this distribution is unknown. Monte Carlo simulations and analytical expressions were used to identify three factors that can transform a unimodal distribution of separations into a bimodal distribution of FRET efficiencies, considering the simplest case in which the donor decay in the absence of acceptor is mono-exponential. These factors are 1) large variation in the donor-acceptor separation, 2) photo-physical transitions of the acceptor between a normal and a dark state, and 3) absence of or slow molecular rotation of the fluorophores with respect to each other. Complex donor decay behavior exhibited by fluorescent proteins in the absence of an acceptor [17]–[19], together with the observation that constructs consisting of a single tandem pair of a covalently attached fluorescent protein donor and acceptor in close proximity do not decay as a single exponential [13], [14], further suggest that not only can these three factors influence FRET between fluorescent protein donors and acceptors but that observed bi-exponential decay behavior in the presence of acceptor when in its absence the donor excited-state decays monoexponentially can arise for reasons other than partial complexation or aggregation of donor- and acceptor-containing moieties.

Even though our study has demonstrated that all three of these factors can give rise to a complex fluorescence decay, it is important to realize that the degree to which they can impact FRET measurements may vary for different samples. A thoughtful evaluation of the fluorophores being used, and the molecules whose interaction is under evaluation is required. Furthermore, the mechanism by which these factors result in bimodal distributions of FRET efficiencies also warrants consideration. To illustrate this, we will consider the impact of these factors on C5V, a broadly used FRET calibration standard [14]. C5V is comprised of a blue FP tethered to a yellow FP by a 5 amino acid linker. When expressed in cells C5V has a high average FRET efficiency (43%) and its fluorescence has a complex multi-exponential decay [14].

### Could *R_DA_* Heterogeneity Contribute to the Complex Excited-state Decay of C5V?

Variation in *R_DA_*, for example a normal distance distribution, creates an increasingly bimodal efficiency distribution as its variance increases because the inverse 6^th^ power dependence of *k_F_* on *R_DA_* (Eq 1) dictates that *R_DA_* values that are much greater than *R_0_* will lead to low FRET efficiencies while much smaller ones will lead to efficiencies close to 1. For the FRET pairs modeled in figure 2, those having *R_DA_* values<3.3 nm (Fig. 2A Yellow tint) will have FRET efficiencies >95% (Fig. 2B Yellow tint). Similarly, pairs with *R_DA_* values >8.8 nm (Fig. 2A Green tint) will have FRET efficiencies <5% (Fig. 2B Green tint). Thus, as *R_DA_* heterogeneity increases, the number of molecules in the population that will have *R_DA_* values that fall into these 2 ranges will increase, generating a bimodal distribution of FRET efficiencies with peak values near 0 and 1 (Fig. 2B).

Some heterogeneity in *R_DA_* is expected in most biological samples, but the extent is typically unknown. The Cerulean and Venus fluorophores in C5V have a rigid ß-barrel structure with dimensions of approximately 2 nm in diameter by 4 nm in length [43], [44]. Their fluorophores are situated roughly at the center of the barrel. The 5 amino acids (AA) C5V linker length distribution is not known, but can be estimated. The distance separating individual amino acids in an α-helix is ∼0.15 nm/AA, and in the more extended ß-sheet it is ∼0.35 nm/AA [45]. Accordingly, the estimated range of *R_DA_* values possible for C5V would be ∼2 nm (close side-by-side contact of the ß-barrels) to 6.1 nm (for two FPs arranged end-to-end, 4 nm if in direct contact, separated by a fully extended 5 amino acid linker, 6×0.35 nm). Basing a Gaussian model for C5V on this, with *R_DA_* = 3.9±1.9 nm (mean±2SD), i.e., with range corresponding to twice the standard deviation and assuming *κ* ^2^ = 2/3, this *R_DA_* distribution (mean±∼25%) would have an average efficiency greater than 0.7, much higher than the 0.43 value observed. Given that the assumptions involved in modeling C5V as a broad Gaussian distribution of *R_DA_* values are valid and appropriate, it appears unlikely that *R_DA_* heterogeneity alone can explain C5Vs complex decay. It is however worth considering that other *R_DA_* distribution models, such as a Lorentzian or one based on end-to-end polymer lengths might be more appropriate for modeling C5V [46], [47]. Furthermore *R_DA_* values and *κ* ^2^ values may be correlated [48], which may add an additional complexity to modeling C5V FRET behavior.

### Could Venus Dark States Contribute to the Complex Excited-state Decay of C5V?

Fluorescent proteins are known to have both bright and dark states, and transition rapidly between these states on a µs [23] or slower [24] time-scale (*Flickering* & *Blinking* respectively). Thus, some fluorophores in a FRET pair might be in the dark state. It has also been suggested that a fraction of FPs fold improperly [49] resulting in a permanent ‘dark’ state. While not explicitly addressing the FRET behavior of a covalently linked donor-acceptor construct like C5V, in FRET studies in which a free donor species can form a complex with an independent acceptor species there will be often fractions that fail to form a complex and therefore will not contribute to FRET [8], [50]. Similarly, when biological macro-molecules are chemically modified with fluorophores these reactions often fail to label every molecule resulting in a fraction of unlabeled donors and/or acceptors [50]. The impact of fractional complex formation and/or fractional labeling on FRET efficiencies measured using sensitized emission has been investigated previously [8], [50] and is analogous to the impact of fluorescent protein flickering, blinking and improper folding observed with FPs. As these phenomena all have dark states that last longer than the excited state lifetime of a FRET donor (typically 2–4 ns), FRET pairs consisting of donors in close proximity to ‘dark’ acceptors will not undergo FRET if dark state FPs cannot act as FRET acceptors. The impact of acceptor dark states, as described above, is depicted in figure 3. As the fraction of acceptors in a dark state increases, the amplitude of the normal distribution of FRET efficiencies is attenuated, and the number of molecules with *E* = 0 increases, thus transforming a normally distributed population of FRET efficiencies into a bimodal distribution.

While it is possible that Venus molecules in a dark state might be responsible for the multi-exponential fluorescence decay observed for C5V, several lines of reasoning suggests that this is unlikely. First, as FP dark absorbers have been described [51], it is possible that dark state FPs can also act as FRET acceptors. Second, while FP flickering has been observed with one photon excitation, it has not been observed with two-photon excitation [52]. The FLIM measurements of C5V were obtained with two-photon excitation [14]. Furthermore, the FRET efficiency for C5V has been measured using both one- and two-photon excitation and both approaches yield similar FRET efficiencies [14] suggesting that flickering activity, if present at all, is at such a low level that it does not influence the measured FRET efficiency. Third, using fluorescence correlation spectroscopy (FCS) and brightness analysis, a GFP-dimer was shown to have twice the brightness of a monomer [52]. This would not be possible if a large fraction of unfolded GFP molecules were present [52]. Like GFP, the brightness of a Venus-dimer was also double the brightness of a Venus-monomer [53]. Brightness analysis cannot, however, eliminate the possibility that flickering on a fast time scale is occurring because if the rate of flickering is faster than the correlation time the brightness of both monomers and dimers would be attenuated to the same extent [52]. Finally, FCS experiments with Venus excited by two-photon excitation also failed to detect any evidence for flickering activity under physiological conditions (neutral pH and low excitation energy; [53]). Thus, it is unlikely that a Venus dark state is responsible for the complex fluorescence decay observed for C5V.

### Could the Large Size of Cerulean and Venus Fluorophore-containing Protein Segments Contribute to the Complex Excited-state Decay of C5V?

A third factor that might give rise to a bi(or multi)modal distribution of C5V FRET, and thereby contribute to the complexity of excited-state decay of C5V is the large size of FPs (28,000 kDa). Since Cerulean and Venus are large, their average rotational correlation times are long, (18.3±1.5) ns and (20.3±1.8) ns respectively) compared to their average excited-state lifetimes (3.18±0.03 and 3.03±0.01 ns) [21]. Furthermore, when FPs are attached to other proteins, as in a FRET experiment, their rotational correlation times will increase [39]. Vis-à-vis FRET, a donor can only transfer energy to a neighbor while it is in the excited state. The probability of FRET is dependent on the relative orientation of the donor and acceptor transition dipoles with respect to each other and to their separation vector (as indicated by *κ* ^2^), and for isotropically or pseudo-isotropically randomly oriented donors and acceptors a majority of molecules in the population will have a low probability for FRET (Fig. 4B). If, during the excited state, donors and acceptors rotate rapidly and completely over independent isotropic or pseudo-isotropic orientational distributions, they will then sample many different *κ* ^2^ values while in the excited state. Under these rapid isotropic sampling conditions an average value for *κ* ^2^ is appropriate, and the probability for transfer will be larger than for the case in which each and every molecule retains a fixed *κ* ^2^ within the random population (this is also true for more limited reorientational freedom, but in that case the dynamic average of the orientation factor will assume the dynamic random average value of 2/3 only under particular circumstances). Since FPs rotate slowly, dynamic isotropic conditions do not apply. Thus, when a given Cerulean molecule is excited, the orientation of its emission dipole, as well as the orientation of any nearby Venus absorption dipoles will change negligibly, *κ* ^2^ will remain constant throughout the excited state lifetime, and little, if any averaging will occur. Accordingly, it is inappropriate, even if isotropic or pseudo-isotropic orientational distributions are approximated (cf. [33]), to assume a *κ* ^2^ value of 2/3 in FRET experiments using FP donors and acceptors. On a molecule-by-molecule basis, *κ* ^2^ can potentially have any value from 0 to 4. If we assume that the orientations of the donor and acceptor dipoles are independently isotropically or pseudo-isotropically random, then the probability of any specific *κ* ^2^ value will be heavily skewed towards values near zero (Fig. 4B), and this may then transform a normally distributed population of separation distances into a bimodal distribution of FRET efficiencies (Fig. 5A). It is worth noting that the impact of having *κ* ^2^ values skewed towards near zero values on the average FRET efficiency for acceptor-donor pairs with large *R_DA_* (relative to the Förster distance) will be less pronounced than for pairs where *R_DA_* is equivalent or smaller than the Förster distance (compare Dynamic and Static curves in Figs. 5C).

Given the slow rotational correlation times of fluorescent proteins (relative to their fluorescent lifetimes) it is likely that energy transfer between FPs occurs in the static regime. Thus, for energy transfer between FPs, assigning a *κ* ^2^ value of 2/3 to transform an experimentally measured FRET efficiency into an estimate of the donor/acceptor separation is flawed. How then can this distance be estimated from FRET measurements? Based on theory [26], [30] and Monte-Carlo simulations presented here, in the static random isotropic orientational regime the average *κ* ^2^ value of an ensemble is dependent on separation. Therefore, if FP donors and acceptors are oriented randomly (the same assumption employed in the dynamic regime using a 2/3 *κ* ^2^ value) the distance between an FP donor and acceptor can be estimated from an experimentally determined FRET measurement. The static curve plotted in figure 5C may be used to estimate separation; an average FRET efficiency value (Y-axis) defines a unique *R_DA_/R_0_* value (X-axis). Multiplying this ratio by the appropriate Förster distance for a specific FRET donor-acceptor pair is an estimate of the separation.

If FP donors and acceptors are rigidly attached to each other, neither static nor dynamic reorientational regimes are relevant as the FRET efficiency would reflect a single species with fixed *R_DA_* and *κ* ^2^ values, and should therefore exhibit a single exponential decay (if the donor alone decays as a monoexponential). For C5V and other tandem FP FRET pairs this is clearly not observed. While the assumption of random orientations is justified for free FPs in solution, it is unclear if this isotropic assumption is appropriate for a covalent tandem FRET pair like C5V, though it does appear to be true for the more complex system of FP-labelled IgEFc [33]. For example, physical restrictions of the linker and the rigid structures of ß-barrels might limit or skew the distribution of possible *κ* ^2^ values. Still, the possibility that static conditions will favor low *κ* ^2^ values for many C5V molecules remains. Thus, the slow segmental rotation of FPs also emerges, along with *R_DA_* heterogeneity, as a plausible contributor to the complex decay of C5V.

Single-pair FRET (spFRET) [54], like FLIM, provides a way of determining the distribution of microscopic FRET efficiencies. Unfortunately, fluorescent proteins are not ideally suited for spFRET studies. Furthermore, the absence of FRET (the so called ‘zero-peak’) in spFRET studies is often attributed to bleaching, even though its true origin is not known [54]. For these reasons, the factors described in this study may have been missed with this approach.

We have shown using Monte-Carlo simulations that *R_DA_* heterogeneity, acceptor dark states, and slow rotation all have the potential to transform a homogeneous distribution of *R_DA_* values into a bimodal distribution of FRET efficiencies. These factors should be considered when interpreting FP decay curves, particularly for dual component analysis. The impact of these factors on **〈** *E* **〉** also indicates that interpreting FP FRET in terms of absolute separations is problematic.

## Methods

### Monte Carlo Simulations

Monte Carlo simulations were performed in Igor Pro (vs 6.22). Each simulation was based on generating 100,000 random replicates. Gaussian *R_DA_* populations were generated using the ***gnoise(n)*** command where ***n*** is the desired standard deviation of the population. Corresponding *k_T_* values were calculated using either Eq 1 (dynamic random isotropic reorientational regime) or Eq 10 (static random reorientational regime), and *k_T_* values transformed into FRET efficiencies using Equation 3. For these simulations *τ_0D_* was set to 3 ns, and the *R_0_* value was 5.4 nm. To generate a distribution of *κ* ^2^ values to simulate an isotropic population of donors and acceptors, 100,000 random values for the angles *θ* and *ω* were generated. First, a uniform distribution of numbers ranging from 0 to 1 was generated using the ***enoise*** command. Next, the inverse cosine of these values was calculated to generate a population of angles expected for an isotropic distribution. These values of *θ* and *ω* were used in Equation 6 to generate the population of *κ* ^2^ values, and these in turn were used in Equation 10 to calculate FRET transfer rate constants in the static regime. To simulate the impact of acceptor dark states, the *k_T_* population was modified by randomly setting ***(*** **1** ***−p)*** of the *k_T_* values to zero using the ***binomialnoise(n,p)*** command where ***n*** was set to 1 and ***p*** was set to the fraction of molecules in the population in a bright state.

### Derivation of Probability Distribution and Density for FRET Efficiency in the Static Random Isotropic Reorientational Regime

The orientation factor, 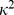, can be written as:
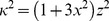
(13)where 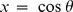 and 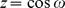 (see Eq 6). In this form, random selection of the values of 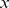 and 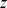 in the intervals −1 to 1 correspond to random selections of values of 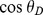
and 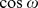, respectively, in the intervals 0 to π when those angles are distributed isotropically (i.e., randomly in three-dimensional space), in which case the probability of finding given angles 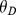 and 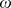 in the distribution is proportional to the sine of those angles. In these terms, the efficiency of energy transfer, 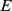, for different values of 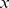 and 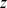 is given by:
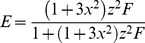
(14)where the factor 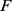 depends on the donor-acceptor distance, RDA, and the Förster separaton when 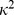 equals 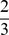, R0, as defined in Eq 15:
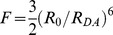
(15)From equation 14 it is seen that 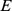 = 0 for 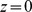,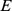 increases from 0 to 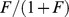 when 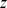 goes from 0 to 1 along the line 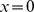, whereas along 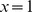 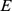 increases from 0 to 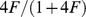 when 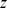 increases from 0 to 1. Intersecting the 2-dimensional curve given by equation 14 at a specific value for 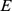 yields a curved line in the 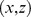-plane given by:
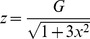
(16)where 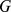 is defined by:



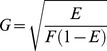
(17)The area below the curve defined by equation 16 inside the square formed by all values of 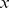 between 0 and 1, and 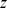 between 0 and 1, represents 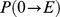, the range probability, that is, the probability that the efficiency has a value between 0 and that specific 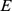-value. Identifying 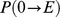 as an integral over the function given in equation 16 and evaluating the integral, yields the following expression for the probability distribution 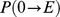:
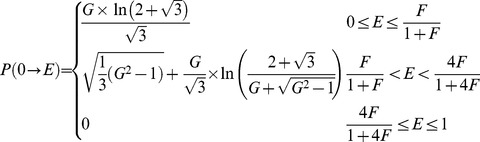
(18)


Differentiating 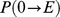 with respect to 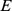 yields 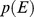 the probability density for the efficiency. In other words, the probability of finding efficiency values between 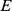 and 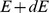 is equal to 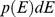, with 
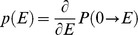
. On performing this differentiation:
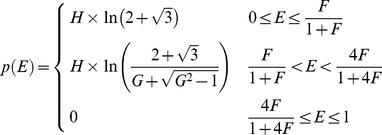
(19)is obtained where 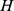 is defined as:



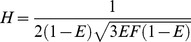
(20)The probability density for the efficiency, 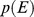, has a maximum value at 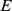 = 0; actually, 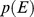 goes to infinity when 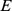 reaches 0. When 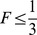, 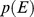 decreases with increasing 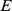 for all values at which 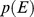 is larger than 0, that is, for 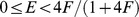; 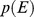 is identically zero, for 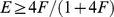. When 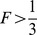, 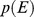 decreases with increasing 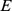 from 0 to 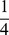, reaches a local minimum of, 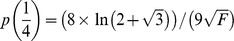, at 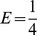, then increases between 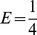 and 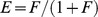, reaching a peak-value equal to 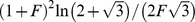 at 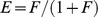, and decreases from that value to 0 as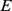 increases from 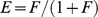 to 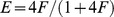.
