# Correction: The Impact of Heterogeneity and Dark Acceptor States on FRET: Implications for Using Fluorescent Protein Donors and Acceptors

**DOI:** 10.1371/annotation/ea42b98e-12b7-4940-8c4e-6142dedbb997

**Published:** 2013-01-14

**Authors:** Steven S. Vogel, Tuan A. Nguyen, B. Wieb van der Meer, Paul S. Blank

There were numerous errors throughout the article. 

The corrections can be viewed here: http://www.plosone.org/corrections/pone.0049593.cn.tif

